# Extended-Spectrum β-lactamase (ESBL) producing *Enterobacter aerogenes *phenotypically misidentified as *Klebsiella pneumoniae *or *K. terrigena*

**DOI:** 10.1186/1471-2180-4-49

**Published:** 2004-12-24

**Authors:** Geert Claeys, Thierry De Baere, Georges Wauters, Patricia Vandecandelaere, Gerda Verschraegen, An Muylaert, Mario Vaneechoutte

**Affiliations:** 1 Department of Microbiology, Ghent University Hospital, Ghent, Belgium; 2 Unit of Medical Microbiology, Université Catholique Louvain, Brussels, Belgium; 3 Department of Microbiology, Jan Yperman Hospital, Ieper, Belgium; 4 Department of Microbiology, Streeklaboratorium Zeeuws-Vlaanderen, Terneuzen, The Netherlands

## Abstract

**Background:**

*Enterobacter aerogenes *and *Klebsiella pneumoniae *are common isolates in clinical microbiology and important as producers of extended spectrum β-lactamases (ESBL). The discrimination between both species, which is routinely based on biochemical characteristics, is generally accepted to be straightforward. Here we report that genotypically unrelated strains of *E. aerogenes *can be misidentified as *K. pneumoniae *by routine laboratories using standard biochemical identification and using identification automates.

**Results:**

Ten clinical isolates, identified as *K. pneumoniae *or *K. terrigena *with the routinely used biochemical tests and with API-20E, were identified as *E. aerogenes *by tDNA-PCR – an identification that was confirmed by 16S rRNA gene sequencing for five of these isolates. Misidentification also occurred when using the automated identification systems Vitek 2 and Phoenix, and was due to delayed positivity for ornithine decarboxylase and motility. Subculture and prolonged incubation resulted in positive results for ornithine decarboxylase and for motility. It could be shown by RAPD-analysis that the *E. aerogenes *strains belonged to different genotypes.

**Conclusions:**

Clinical *E. aerogenes *isolates can be easily misidentified as *Klebsiella *due to delayed positivity for ornithine decarboxylase and motility. The phenomenon may be widespread, since it was shown to occur among genotypically unrelated strains from different hospitals and different isolation dates. A useful clue for correct identification is the presence of an inducible β-lactamase, which is highly unusual for *K. pneumoniae*. In several instances, the use of genotypic techniques like tDNA-PCR may circumvent problems of phenotypic identification.

## Background

Enterobacteriaceae with β-lactam resistance due to the production of Extended-Spectrum β-Lactamases (ESBL) were discovered in the eighties and since that time became epidemic and endemic in hospitals worldwide [1]. Since two decades, 20 to 40 ESBL-producing strains are isolated monthly in our hospital. Amongst the clinical isolates from our hospital, two new TEM-β-lactamase genes were described [2]. In Belgium, as well as in other countries, a shift occurred from *Klebsiella pneumoniae *isolates as the predominant ESBL-producers [3] to predominance of *Enterobacter aerogenes*. It is also known that most of the *E. aerogenes *isolates in the Belgian hospitals belong to one of two predominant Belgian clones (BEI and BEII) [4], a situation which is comparable to that in other countries [5]. During the last years however, we found that several isolates that were identified as *K. pneumoniae *or *K. terrigena *by conventional biochemical testing were in fact *E. aerogenes *as could be shown by the use of genotypic methods, i.e. tDNA-PCR, validated by 16S rRNA gene sequencing, and by extensive phenotypic testing (including subculture and prolonged incubation).

## Results

For ten clinical isolates, i.e. seven ESBL-producing clinical isolates collected during 2001 and three more recently collected isolates (Table 1), the API20E codes 5205773 (isolates GA1, GA2, GA3, MN2, MN3 and VGM), 5205753 (isolates DHJ1 and DHJ3) or 5204673 (isolates RA and DBH) were obtained. The first code resulted in a weak identification as either *E. aerogenes*, *K. pneumoniae *or *Raoultella *(*Klebsiella*) *planticola*, the second code did not yield any identification, and the third code resulted in a weak but acceptable identification as *K. terrigena*. The isolates with the codes 5205773 and 5205753 were identified as *K. pneumoniae *by additional biochemical testing due to negative reactions for motility (tested in semi-solid agar) and ornithine decarboxylase. However, these isolates all possessed an inducible cefalosporinase, as detected on the antibiogram using a disk approximation test, a finding which strongly contradicts an identification as *K. pneumoniae *or *Klebsiella *sp.

In fact, the first hint that these strains, phenotypically identified as *K. pneumoniae*, were actually *E. aerogenes*, came from tDNA-PCR based identification. Using this method, all isolates were identified as *E. aerogenes*, and this by comparison of the obtained fingerprint – composed of amplified intergenic tRNA spacers of 101, 106, 111, 115, 121, 189, 190, 198 or 289, and 391 bp in length – with a library containing fingerprints of more than 3000 strains belonging to hundreds of species, available at .

Confirmation of this genotypic identification was obtained by 16S rRNA gene sequencing for five isolates (Table 1). Analysis yielded a similarity of between 99.8% and 100% to *E. aerogenes *Genbank entries.

The presence of a genuine *K. pneumoniae *isolate in patient DHJ (Table 1) further complicated the identification.

This observation lead us to carry out additional phenotypic testing. Using the hanging drop method for testing motility, a few motile cells were observed, and upon retesting in semi-solid medium, weak migration could be observed. Like most biochemical tests in the routine laboratory, ornithine decarboxylase is read after overnight or 24 hours of incubation, but when the incubation period was prolonged to up to 2–5 days, all isolates tested positive.

Because at present automated systems are frequently used for routine identification, a selection of six strains, containing four isolates of the study and two controls, i.e. phenotypically correctly identified *E. aerogenes *(LBV268) and *K. pneumoniae *(BG) isolates, were tested in two different systems, i.e. Vitek 2 (bioMérieux, Marcy l'Etoile, France) and Phoenix (BD Biosciences, Sparks, Md.). Both automated systems yielded the same results as the API20E, i.e. that the *E. aerogenes *isolates, aberrant due to a slow reaction for motility and ornithine decarboxylase, were misidentified as *K. pneumoniae *(Table 1). This misidentification by the automated systems is not unexpected, since they are based on biochemical testing only and a reading time of 24 hours or less. The control strains were correctly identified.

Disk diffusion antibiotic susceptibility testing, carried out according the NCCLS guidelines, revealed basically the same resistotype for all isolates, characterized by resistance to ceftazidime and susceptibility to ceftriaxone. Additional resistance to aztreonam was observed for some isolates, reflecting the most dominant resistance patterns for the *E. aerogenes *isolates in our hospital. All isolates were also found to carry high-level resistance to cefoxitin, which is highly unusual for *Klebsiella *spp. Furthermore, the disk-approximation test with an amoxycillin-clavulanic acid disk close to β-lactam disks on Mueller-Hinton II agar, showed a combined pattern of synergy (broadening of the inhibition zone in the direction of clavulanic acid) and antagonism (flattening of the inhibition zone), which is suggestive for a combination of an ESBL and an inducible β-lactamase. Again, inducible β-lactamases are very rare in *Klebsiella *spp. but typical for *Enterobacter *spp. It should be noticed that this phenomenon will not be detected by automated MIC-determination systems like Vitek 2 and Phoenix. Using PCR and sequencing as described previously [2], the presence of TEM-5 could be shown in the isolates of patients GA and MN, and SHV-4 in the isolate of patient DHJ.

**Figure 1:**
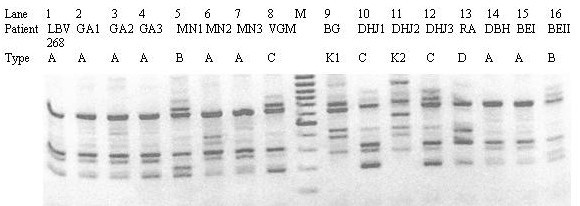
RAPD analysis of *Enterobacter aerogenes *and *Klebsiella pneumoniae *strains used in this study Lane M: DNA molecular weight marker (100 base pair ladder). The *E. aerogenes *RAPD-types are indicated as A, B, C and D and the *K. pneumoniae *types are indicated as K1 an K2. RAPD-type A corresponds to clone BEI, type B to clone BEII, type C to BEII-related clone and type D presents a strain unrelated to clones BEI and BEII. Negative image of ethidium bromide stained agarose electrophoresis

The genotypic relationship of the phenotypically aberrant isolates was investigated with AP-PCR. Isolates GA1, GA2, GA3, MN2, MN3 and DBH were corresponding to Belgian clone BEI (Figure 1, pattern A), isolates VGM, DHJ1 and DHJ3 were closely related to Belgian clone BEII (Figure 1, pattern B, differing from pattern C, characteristic of clone BEII, by a single extra band) while isolate RA was not related to any of the others (Figure 1, pattern D). This genetic diversity among the phenotypically aberrant strains makes it probable that strains with this kind of aberrant phenotype are not restricted to a single clone within *E. aerogenes*.

## Discussion

*K. pneumoniae *and *E. aerogenes *are taxonomically closely related species [6] which share many characteristics. Our sequencing results (unpublished) and those of others [6] confirm that the genus *Enterobacter *is polyphyletic and that *E. aerogenes *should be placed within the genus *Klebsiella*. However, differentiation between *E. aerogenes *and *K. pneumoniae *is usually straightforward when based on testing for ornithine decarboxylase and motility, both positive for *E. aerogenes*. This is also reflected in the name "*Klebsiella mobilis*" [7], which is known as a valid synonym for *E. aerogenes*. Apparently, in some *E. aerogenes *isolates, the expression of these characteristics can be weak and/or delayed, and these are therefore scored negative when reading is done after the incubation periods that are routinely applied (overnight – 24 hours).

However, the combination of an inducible β-lactamase and/or high-level cefoxitin resistance, which are rare in *Klebsiella *spp., and an identification as *Klebsiella *sp. should warrant further investigation.

The phenomenon of *E. aerogenes *misidentified as *K. pneumoniae *or *K. terrigena *due to delayed or negative ornithine decarboxylase and motility was reported previously, and was also discovered because of unexpected imipenem resistance of the so-called *K. pneumoniae *isolates [8]. Also in this case, subculture and prolonged incubation restored the positivity for these characteristics.

The problem of misidentification of *E. aerogenes *as *K. pneumoniae *(or even *K. terrigena*) is probably not uncommon and probably also geographically widespread. This can be deduced from the following considerations: i) this phenomenon of misidentification of *E. aerogenes *was already reported in 1993 [8], ii) the phenomenon occurred in genotypically different organisms, iii) the isolates were found over an extended period of time – also recently, and finally iv) we received similar strains from other Belgian hospitals (unpublished data). It should be noted that misidentification also occurred when using the newer and automated systems like Vitek2 and Phoenix.

On the other hand, the problem seems to be largely unknown. In a recent study, Hansen and colleagues [9] carried out an interlaboratory comparison of the efficacy of 18 biochemical tests for the identification of 242 strains of different *Klebsiella *species and of *Enterobacter aerogenes*, but do not mention the problem of possible delayed activity, possibly also because the study started from validated strains of each species.

## Conclusions

Identification in a routine clinical microbiology laboratory of the most commonly encountered Enterobacteriaceae is usually considered to be fast and straightforward, but apparently identification problems may occur due to diminished or delayed expression of some characteristics, even for well-established species like *E. aerogenes *and *K. pneumoniae*. Here we showed that *E. aerogenes *isolates exist for which ornithine decarboxylase and motility are negative or delayed positive, and that as such these isolates can be misidentified as *K. pneumoniae*. This phenomenon may be quite frequent and geographically widespread. Genotypic identification techniques like tDNA-PCR, which moreover are cheaper than phenotypic testing for many bacterial species, can be semi-automatized, are faster and mostly have a higher discriminatory power, which is also reflected in this study.

## Methods

### tDNA-PCR

tDNA-PCR was carried out using the outwardly directed tRNA-gene consensus primers T5A (5'AGTCCGGTGCTCTAACCAACTGAG) and T3B (5'AGGTCGCGGGTTCGAATCC), thus amplifying the intergenic tRNA-spacers, as described previously [10,11]. Electropherograms were normalized using GeneScan Analysis software, version 2.1 (Applied Biosystems). Transformation of GeneScan tables (ABI310, McIntosh) to tables on IBM, separation into separate digital fingerprints, and comparison of the digital tDNA-PCR fingerprints with a library of tDNA-PCR-fingerprints obtained from a large collection of reference strains, was done using in house software described previously [10].

### 16S rRNA gene sequencing

For five of the phenotypically aberrant isolates, the complete 16S rRNA sequence was determined by amplification of the 16S rRNA-gene with the primers 5'-AGTTTGATCCTGGCTCAG and 5'-TACCTTGTTACGACTTCGTCCCA [12], and sequencing was performed as described previously [12]. Comparison of the obtained 16S rDNA-sequence with all known sequences in Genbank was carried out using the BLAST software (National Center for Biotechnology Information ).

### RAPD-analysis for strain typing

The genotypic relationship of the isolates was investigated using arbitrarily primed PCR with RAPD Ready-to-Go beads (Amersham Pharmacia, Uppsala, Sweden) and the ERIC II primer 5'-AAGTAAGTGACTGGGGTGAGCG [13]. Analysis of the fingerprints was obtained by visual interpretation on ethidium bromide stained electrophoresis gels.

## Authors' contributions

GC, GV and AM were responsible for sample collection and initial biochemical identification. PVD and GW carried out automated biochemical identification. GW in addition carried out extended biochemical characterization. TDB and MV carried out the molecular analysis. GC, GV, TDB and MV drafted the manuscript. All authors read and approved the final manuscript.

## Figures and Tables

**Table 1:** Clinical data and phenotypic and genotypic identification results of the *Klebsiella pneumoniae *and *Enterobacter aerogenes *isolates used in this study

**Strain**	**Sample**	**Motility^b^**	**Ornithine decarboxylase^b^**	**API Code**	**API Identification^c^**	**Vitek2^d ^Phoenix^d^**	**tDNA-PCR^e^**	**Clone^f^**
Group 1								
LBV268		+	+	5305773	*E. aerogenes*	*E. aerogenes*	*E. aerogenes*	BEI
MN1	Aspirate	+	+	5305773	*E. aerogenes*	NT	*E. aerogenes*	BEII
BEI 166		+	+	5305773	*E. aerogenes*	NT	*E. aerogenes*	BEI
BEII 169		+	+	5305773	*E. aerogenes*	NT	*E. aerogenes*	BEII
Group 2								
BG	Blood culture	-	-	5005763	*K. pneumoniae*/*K. terrigena*	*K. pneumoniae*	*K. pneumoniae*	K1
DHJ2	Aspirate	-	-	5215773	*K. pneumoniae*	*K. pneumoniae*	*K. pneumoniae*	K2
Group 3								
GA1	Wound	(-)	(-)	5205773	Weak	NT	*E. aerogenes*	BEI
GA2	Urine	(-)	(-)	5205773	Weak	NT	*E. aerogenes*	BEI
GA3	Urine	(-)	(-)	5205773	Weak	*K. pneumoniae*	E. aerogenes*	BEI
MN2	Ascites	(-)	(-)	5205773	Weak	*K. pneumoniae*	*E. aerogenes*	BEI
MN3	Urine	(-)	(-)	5205773	Weak	NT	E. aerogenes*	BEI
VGM	Sputum	(-)	(-)	5205773	Weak	*K. pneumoniae*	*E. aerogenes*	BEII-related
DHJ1	Urine	(-)	(-)	5205753	No identification	NT	E. aerogenes*	BEII-related
DHJ3	Blood culture	(-)	(-)	5205753	No identification	NT	*E. aerogenes*	BEII-related
RA	Throat isolate	(-)	(-)	5204673	*K. terrigena*	NT	E. aerogenes*	Non-related
DBH	Urine	(-)	(-)	5204673	*K. terrigena*	NT	E. aerogenes*	BEI

a: Data for genuine *E. aerogenes *isolates are presented first (group 1). Clinical isolate LBV268 was used as control for analysis on automated phenotypical identification systems. The isolates BEI 166 and BEII 169 were shown previously [4] to belong to the two major *E. aerogenes *clones (BEI and BEII) in Belgium. Group 2 presents data for genuine *K. pneumoniae*. Clinical isolate BG was used as control for analysis on automated phenotypical identification systems. The third group presents the data for the phenotypically aberrant *E. aerogenes *isolates.

b: +, positive; -, negative; (-), negative after standard incubation time, only positive after subculturing and/or retesting with prolonged incubation periods.

c: Weak: Weak identification with possibilities: *E. aerogenes*, *K. pneumoniae *or *R. planticola*.

d: NT: not tested.

e: * indicates that identification was confirmed by 16S rDNA sequence analysis.

f: Clonal relationships were determined using RAPD-analysis.
